# A morphological analysis of the modern human frontal bone from Hahnöfersand, Germany

**DOI:** 10.1038/s41598-026-48468-5

**Published:** 2026-04-17

**Authors:** Carolin Röding, Antonio Profico, Michael Merkel, Katerina Harvati

**Affiliations:** 1 Paleoanthropology, Senckenberg Centre for Human Evolution and Palaeoenvironment, Eberhard Karls University of Tübingen, Rümelinstr. 23, 72070 Tübingen, Germany; 2 Institute for Archaeological Sciences, Eberhard Karls University of Tübingen, Rümelinstr. 23, 72070 Tübingen, Germany; 3 Department of Biology, University of Pisa, Lungarno Pacinotti 43, 56126 Pisa, Italy; 4 Archäologisches Museum Hamburg und Stadtmuseum Harburg, Museumsplatz 2, 21073 Hamburg, Germany; 5 DFG Centre of Advanced Studies ‘Words, Bones, Genes, Tools, Eberhard Karls University of Tübingen, Rümelinstr. 23, 72070 Tübingen, Germany; 6 HUMAN ORIGINS – Cluster of Excellence for Integrative Human Origins Studies (EXC 3101), Eberhard Karls University of Tübingen, Rümelinstr. 23, 72070 Tübingen, Germany

**Keywords:** Neanderthal, Human evolution, Surface registration, Virtual anthropology, Anatomy, Evolution

## Abstract

Frontal bone morphology is considered informative concerning phylogenetic and taxonomic affinities in hominin fossils. However, evaluating individual frontal bone features, especially macroscopically and with qualitative data, can produce contradictory results. One example is the enigmatic frontal bone from Hahnöfersand, Germany, initially dated to ca. 36 ka. A previous description of this specimen found that it exhibits a mix of modern human- and Neanderthal-like features and interpreted it as a potential hybrid between *Homo neanderthalensis* and *Homo sapiens*. In this case study, we apply a nearly landmark-free technique (i.e., surface registration) to reassess Hahnöfersand’s supposed intermediate morphology in the context of a revised chronology of ca. 7.5 ka. A three-dimensional comparative analysis was conducted using a dataset of Neanderthals, Middle Pleistocene European hominins, and a diverse *Homo sapiens* sample. Principal components analysis, Mahalanobis distances, and pairwise Procrustes distances place Hahnöfersand within the variability of Holocene *Homo sapiens*, with no evidence of intermediate morphology. These findings support Hahnöfersand’s attribution to recent *Homo sapiens* and highlight the efficacy of the surface registration method for morphological assessments of taxonomy in isolated fragmentary remains, where interpretation is driven entirely by the preserved morphology and, if available, dating.

**Supplementary Information:**

The online version contains supplementary material available at 10.1038/s41598-026-48468-5.

## Introduction

**Fig. 1:**
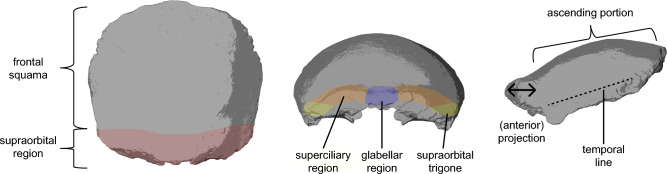
Illustration of key terminology. Illustrations were made based on the anatomical area captured in our dataset, i.e., the entire reconstructed external morphology of the frontal bone from Hahnöfersand (cf. Materials and Methods, Figure 2).

Frontal bone morphology is important in the definition and diagnosis of various Pleistocene hominin groups (e.g.,^1–6^), and often features prominently in the description of hominin fossils (e.g.,^7–13^). For example, a vertical, high frontal squama, a clear delimitation between the superciliary region and the lateral portions of the supraorbital region, as well as a reduced trigone (the most lateral part of the supraorbital region, e.g.,^14,15^; see Fig. 1 for illustrations of key terminology) are viewed as typical features of *Homo sapiens* (e.g.,^16–18^). In contrast, a flat frontal squama and prominent projecting supraorbital region with both central and lateral elements fused into a continuous supraorbital torus are characteristic of Neanderthals (here defined as *Homo neanderthalensis* individuals dating to MIS7-3) and Middle Pleistocene specimens from Europe and Africa (e.g.,^12,19–21^).

**Fig. 2:**
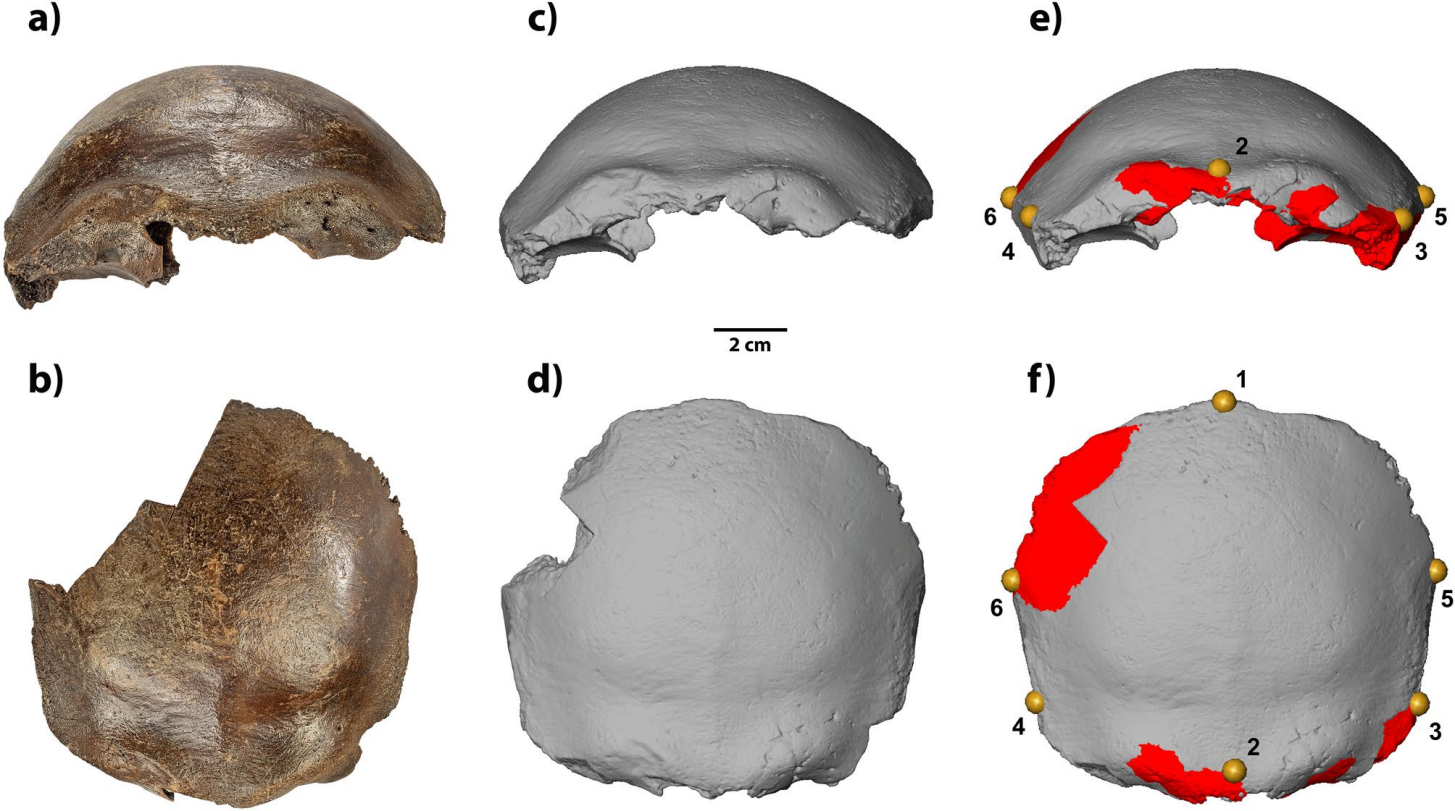
The frontal bone from Hahnöfersand. **A**) and **b**) show the fossil in its current state, **c**) and **d**) a three-dimensional model of the surface scan generated in the early 2000s, and **e**) and **f**) a virtual reconstruction of the frontal bone based on the surface scan with reconstructed elements shown in red. In addition, **e**) and **f**) illustrate the landmark set used in the initial registration of the surface registration method. Numbers refer to landmark definitions provided in Supplementary Table S2. In the upper row, the frontal bone is shown in anterior view (**a**, **c**, **e**), and in the bottom row in superior view (**b**, **d**, **f**). Additional views are shown in Supplementary Figures S1 and S2.

In March 1973, during the construction of a dyke, sediments from the Elbe River near Hahnöfersand, Germany, yielded a well-preserved but enigmatic frontal bone lacking a secure context (Fig. 2, Supplementary Figures S1, S2;^16,22^). A detailed description of this adult specimen was undertaken by Bräuer^16^, who described the frontal squama as relatively flat while admitting to uncertainties regarding its correct orientation when compared to other samples. Although Bräuer found Hahnöfersand’s midsagittal outline to be Neanderthal-like, he described its supraorbital region as showing a clearly delimited superciliary region and a relatively small supraorbital trigone, aligning it with *H. sapiens*. His linear measurements and indices indicated an intermediate morphology between *Homo neanderthalensis* and *H. sapiens* ^16,23^.

Bräuer ^16^ reported a direct radiocarbon date of ca 36 ka uncal. B.P. (Fra-24; but see^22^ for discussion of this now considered falsified date) leading him to interpret the proposed intermediate morphology as potentially indicating a hybrid between *H. neanderthalensis* and *H. sapiens*. This interpretation was repeatedly questioned since, and Hahnöfersand has since been described as a robust *H. sapiens* with some primitive or even Neanderthal-like features, but probably not representing a hybrid (e.g.,^24–29^). Its large size and prominent glabella have been interpreted to indicate male biological sex (^16,22^; but see^30^ for a female attribution).

Bräuer’s^16^ initial interpretation was ultimately rejected based on a new direct date obtained from the specimen, which yielded an age of ca. 7.5 ka and therefore attributed the specimen to the Mesolithic period (OxA-10306;^22^). Thus, Terberger et al.^22^ proposed that the Hahnöfersand individual represents a modern human exhibiting a more extreme than average frontal bone morphology, but still within the expected variation of its time. Nevertheless, Hahnöfersand still occasionally appears in the literature as a potential Neanderthal-modern human hybrid (e.g.,^31^), likely because its new date and revised interpretation were published in German and a format not easily accessible to all international researchers. Hahnöfersand is therefore an example of fragmentary Holocene *H. sapiens* fossils that have been misidentified as Pleistocene finds due to their macroscopically robust morphology and/or assumed context (cf.^29,32–36^). Such cases offer important opportunities to advance both our understanding of intragroup variation and our methodological toolkit, ultimately enabling better analysis of isolated fossil remains from periods when (potentially) multiple hominin groups were present in the same region.

Here, we use the comparative shape analysis of the Hahnöfersand frontal bone as a case study to investigate 1) the degree of agreement in the taxonomic assessment of a reportedly intermediate morphology between traditional methodologies and the surface registration method, and 2) the influence of subsampling in the process of the surface registration method on the results. The subsampling step is essential to reduce the amount of collected data and variables for subsequent analyses, thereby making the surface registration method more versatile in the study of hominin remains.

## Results

Traditionally, frontal bone morphology has been studied via visual observation and comparison of individual morphological traits, but also conventional measurements such as linear distances, angles or ratios, and outlines (e.g.,^16,26,37–41^). More recently, landmark and semi-landmark coordinate-based geometric morphometric analyses have provided additional insights (e.g.,^11,12,42–48^). In contrast to previous approaches, the recently described surface registration method is an almost landmark-free approach that allows us to study the entire preserved morphology as a set of coordinates underlying mesh vertices that represent an object’s surface geometry more fully^49,50^. Thereby, surface areas outside the area delimited by the landmarks, which are used in an initial registration step but excluded from the final dataset, can also be included in the analysis (cf. Materials and Methods: Measurement Protocol, Fig. 1 and 2e, f). This enables a more comprehensive evaluation of even fragmentary specimens, such as the one examined here. To address our above-described objectives, datasets comprising Middle Pleistocene hominins from Europe, *H. neanderthalensis*, and a diverse *H. sapiens* sample were created (cf. Table 1; for detailed information, see Supplementary Table S1).

**Table 1:** Sample overview. More detailed sample description in Supplementary Table S1.

Species	Groups based on dating / associated archaeology		Individuals (abbreviation)
Homo sapiens	Holocene	Mesolithic	Hahnöfersand (Ha)
			Drigge (Dr); Groß Fredenwalde 3 (GF3); Hohlenstein 5830 A (Ho)
		Neolithic (Linear Pottery Culture)	14 individuals from Schwetzingen & Talheim
		Medieval (ca. 600-900 AD)	7 individuals from Nusplingen
			Heidelberg (He)
	Late Pleistocene		Cioclovina (Ci); Grotte des Enfants 6 (GdE6); Mladeč 1 (Ml1); Mladeč 5 (Ml5); Předmostí 3 (Pr3); Předmostí 4 (Pr4); Qafzeh 9 (Qa9); Skhul 5 (Sk5)
Homo neanderthalensis	Late Pleistocene		Amud 1 (Am1); Guattari 1 (Gu1); La Chapelle (LC); La Ferrassie (LF); Neandertal (Ne); Shanidar 1 (Sh1); Shanidar 5 (Sh5); Spy 1 (Sp1)
Middle Pleistocene Europe			Arago 21 (Ar21); Petralona (Pe); Sima de los Huesos 5 (Si5)

### The effect of subsampling

Surface registration, unlike landmark-based analysis, does not depend on a predefined set of landmarks. Instead, it uses an iterative process to adapt a reference mesh, defined by an arbitrary number of vertices, to each specimen in the study sample. For this reason, we compared Principal Component Analysis (PCA) results of multiple datasets with and without subsampling of mesh vertices during the surface registration method (objective 2, for detailed information, see Materials and Methods: Determining the Optimal Subsample). The Pearson correlation coefficients for the baseline, established through multiple iterations without subsampling, range between 0.99947 and 1. Subsampling to 500, 250, 200, 50, and 25 vertices notably altered the PCA results compared to this baseline (Supplementary Figure S3). In contrast, subsampling to 150 and 100 vertices fell within the range of the baseline, with r = 0.99978 and r = 0.99983, respectively. Consequently, 100 vertices were considered an optimal subsample for maximal data reduction without substantial information loss during the k-means clustering (Supplementary Figure S3b).

### Principal component analyses

**Fig. 3:**
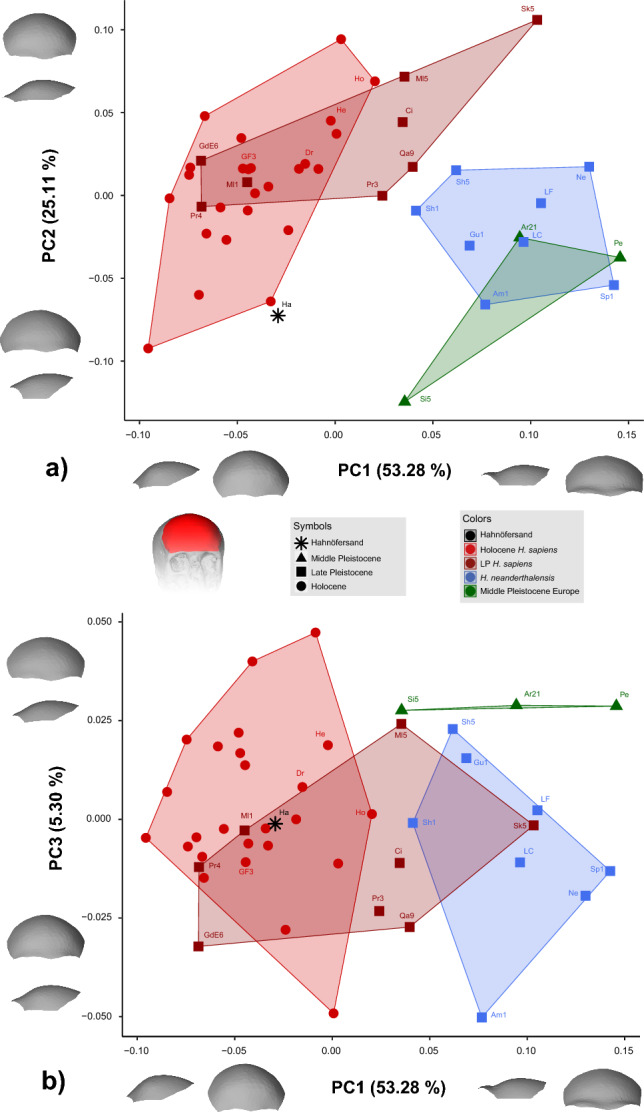
Shape PCA with subsampling to 100 vertices – a) PC1 against PC2 and b) PC1 against PC3. Shape changes along PC axes are illustrated as warped surfaces at ± 2 standard deviations for each PC. Information about the sample and abbreviations of all fossil individuals are listed in Table 1 and Supplementary Table S1.

**Fig. 4:**
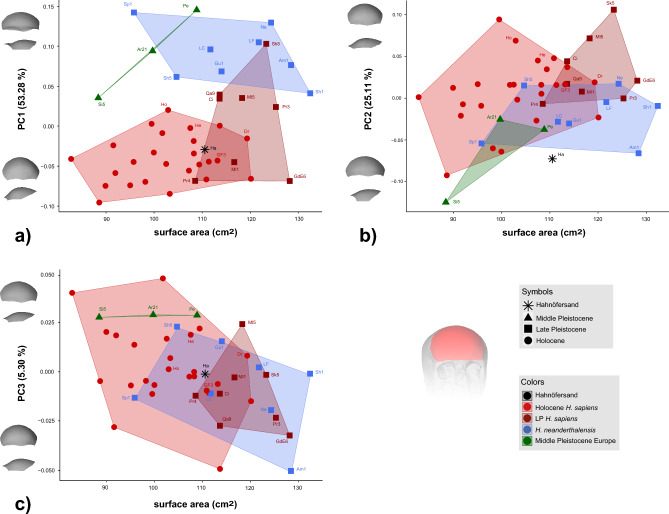
Allometric signal on a) PC1, b) PC2, and c) PC3. Frontal bone size is expressed as surface area (cm^2^). Shape variations are reported at ± 2 standard deviations for each PC. Information about the sample and abbreviations of all fossil individuals are listed in Table 1 and Supplementary Table 1.

The projection of Hahnöfersand into a PCA calculated based on the comparative sample does not alter the results (cf. Fig. 3a, Supplementary Figure S4). Therefore, only the results of the dataset subsampled to 100 vertices without projection of Hahnöfersand are discussed (PCA: Fig. 3; Static Allometry: Fig. 4, Table 2; Procrustes Distances: Table 3; Mahalanobis Distances: Supplementary Table S3) to evaluate the taxonomic affinities of Hahnöfersand (cf. objective 1).

**Table 2:** Pearson correlation tests between size, here log surface area, and shape, here PCs.

**PC**	**Degrees of Freedom**	**t-value**^**1**^	**Correlation Coefficient (R**^**2**^**)**^**1**^	**Regression Slope**^**1**^	**Residual Standard Error of the Regression**^**1**^	**p-value**^**1**^
1	43	0.026	0.331	1.861e-5	0.064	**0.026**
2	43	1.917	0.281	1.083e-5	0.044	0.062
3	43	-3.258	-0.445	-78.91e-5	0.019	**0.002**

PCs derived from the dataset with sub sampling to 100 vertices but without the projection of Hahnöfersand. Significant p-values (α ≤ 0.05) are marked in bold. ^1^rounded to three decimals.

**Table 3:** Pairwise Procrustes Distances (PD) between Hahnöfersand and all *Homo sapiens* (Holocene N=25, Late Pleistocene N=8), Neanderthals (N=8), and Middle Pleistocene Europeans (N=3).

Species / Group		Individuals (Abbreviations)^**1**^	Pairwise PD^**2,3**^	Mean^**3**^	Standard deviation^**3**^
Homo sapiens	Holocene	**Nusplingen 1122**	**0.058**	0.105	0.027
		**Nusplingen 1106a**	**0.064**		
		**Nusplingen 1119**	**0.080**		
		Schwetzingen 4	0.081		
		Schwetzingen 122	0.083		
		Schwetzingen 43	0.087		
		Schwetzingen 67	0.088		
		Schwetzingen 109	0.090		
		Nusplingen 1111a	0.091		
		Groß Fredenwalde 3 (GF3)	0.097		
		Nusplingen 1125	0.099		
		Schwetzingen 14	0.100		
		Schwetzingen 5	0.101		
		Talheim 83_12_2	0.102		
		Drigge (Dr)	0.103		
		Nusplingen 1106b	0.104		
		Talheim 84_2	0.110		
		Nusplingen 1124	0.113		
		Schwetzingen 119	0.113		
		Schwetzingen 48	0.119		
		Heidelberg (He)	0.127		
		Talheim 83_8	0.137		
		Schwetzingen 101	0.138		
		Hohlenstein 5830 A (Ho)	0.159		
		Talheim 83_22_E	0.176		
	Late Pleistocene	Předmostí 4 (Pr4)	0.091	0.135	0.046
		Předmostí 3 (Pr3)	0.099		
		Mladeč 1 (Ml1)	0.099		
		Grtte des Enfantes 6 (GdE6)	0.118		
		Qafzeh 9 (Qa9)	0.130		
		Cioclovina (Ci)	0.141		
		Mladeč 5 (Ml5)	0.173		
		Skhul 5 (Sk5)	0.228		
Homo neanderthalensis		Shanidar 1 (Sh1)	0.105	0.145	0.030
		Guattari 1 (Gu1)	0.118		
		Amud 1 (Am1)	0.126		
		Shanidar 5 (Sh5)	0.136		
		La Chapelle (LC)	0.145		
		La Ferrassie (LF)	0.157		
		Spy 1 (Sp1)	0.182		
		Neandertal (Ne)	0.190		
Middle Pleistocene Europe		Sima de los Huesos 5 (Si5)	0.104	0.148	0.043
		Arago 21 (Ar21)	0.149		
		Petralona (Pe)	0.190		

PDs based on Procrustes coordinates of the subsampled dataset with 100 vertices. The three smallest pairwise PDs to Hahnöfersand are marked in bold. ^1^as listed in Table 1 and Supplementary Table S1^2^Sorted from minimum to maximum within each species / group ^3^rounded to three decimals.

PC1 explains 53.28% of the variance and summarizes shape changes from a relatively high and convex frontal squama with the supraorbital region being a continuation of this curvature (negative PC1 scores); to a relatively low and receding frontal squama with the supraorbital region being clearly delimited from this curvature and projecting anteriorly (positive PC1 scores). PC1 separates Middle Pleistocene individuals from Europe and *H. neanderthalensis* (more positive PC1 scores) from Holocene *H. sapiens* (Fig. 3). Within *H. sapiens*, the geologically older individuals from the Late Pleistocene are, on average, placed at more positive values of PC1 than individuals from the Holocene and thereby partially overlap with our Middle Pleistocene European and Neanderthal samples. This overlap is most pronounced for Skhul V, one of the geologically oldest *H. sapiens* in our sample. Hahnöfersand falls within the Holocene *H. sapiens* variation and exhibits a PC1 score similar to those of the Mesolithic individuals from Drigge and Groß Fredenwalde 3.

PC2 explains 25.11% of the variance and summarizes shape changes from a relatively broad frontal squama with a short ophryon to bregma chord and short temporal lines located low on the cranium (negative PC2 scores); to a relatively narrow frontal squama with a long ophryon to bregma chord and long temporal lines situated high on the cranium (positive PC2 scores). Along PC2, all groups show substantial overlap, except for Middle Pleistocene individuals from Europe (more negative PC2 scores) and Late Pleistocene *H. sapiens* (more positive PC2 scores; Fig. 3a). Hahnöfersand exhibits a negative PC2 score towards the extreme variation of Middle Pleistocene Europeans and Holocene *H. sapiens*.

Only PC3 partially separates our groups among lower PCs, with Middle Pleistocene individuals from Europe showing more positive PC3 scores than Neanderthals (Fig. 3b). PC3 explains 5.30% of the variance and summarizes a combination of relatively subtle shape changes from a relatively narrow and high frontal squama with acute angles between the ophryon to bregma chord and temporal lines (negative PC3 scores); to a relatively broad and low frontal squama with an ophryon to bregma chord parallel to the temporal lines (positive PC3 scores). Hahnöfersand falls within the overlapping variation of Late Pleistocene and Holocene *H. sapiens* and Neanderthals.

The combination of PC1 and PC2 completely separates our *H. sapiens* samples from the overlapping Middle Pleistocene Europeans and Neanderthals. Within *H. sapiens*, the geologically older individuals show a more intermediate position between the Holocene *H. sapiens* sample and the European Middle Pleistocene and Neanderthal samples (Fig. 3a). In contrast, the combination of PC1 and PC3 slightly separates the Middle Pleistocene individuals from Europe from all other groups while showing an overlap between our Late Pleistocene *H. sapiens* and Neanderthal samples (Fig. 3b). The latter is caused by Skhul V and Shanidar 1 plotting in the Neanderthals and Late Pleistocene *H. sapiens* convex hulls, respectively. For the combination of the first three PCs, Hahnöfersand falls close to the Holocene *H. sapiens* variation and plots away from our Neanderthal and European Middle Pleistocene samples (Fig. 3; for an interactive 3D scatter plot see Supplementary Figure S5).

### Static allometry

Middle Pleistocene Europeans and Holocene *H. sapiens* show, on average, smaller frontal bones than Late Pleistocene *H. sapiens* and Neanderthals (Fig. 4). Neanderthals and Holocene *H. sapiens* exhibit significant variation in frontal bone size and overlap with Late Pleistocene *H. sapiens* and Middle Pleistocene Europeans. Hahnöfersand’s frontal bone size falls into the overlap between Late Pleistocene and Holocene *H. sapiens* and Neanderthals.

PC1 and PC3 show significant but weak allometric relationships (PC1: R^2^ = 0.331, p = 0.026; PC3: R^2^ = -0.445, p = 0.002), while PC2 does not exhibit substantial static allometry (R^2^ = 0.281, p = 0.062; Table 2). For PC1, the allometric relationship is driven by the sample size of our, on average, smaller Holocene *H. sapiens*, but there is no clear pattern of intraspecific allometry (Fig. 4a). In contrast, along PC3, most groups show an allometric trend (Fig. 4c). Following this, on average, larger individuals exhibit more negative PC3 scores and show a relatively narrow and high frontal squama with acute angles between the ophryon to bregma chord and temporal lines. In contrast, on average, smaller individuals show a relatively broad and low frontal squama with an ophryon to bregma chord parallel to the temporal lines. While Shanidar 1, the largest individual in our sample, and the Middle Pleistocene Europeans deviate from the trend along PC3, Hahnöfersand follows the expected allometric relationship.

### Morphological distances

Pairwise Procrustes Distances (PD) were used to compare the overall shape of Hahnöfersand to each individual in the comparative dataset. Pairwise PDs differed in the higher decimals, but the overall pattern of results was not influenced by subsampling to 100 vertices. Hahnöfersand is closest in overall shape to three Medieval *H. sapiens* from Germany (Table 3). In our comparative sample, the Late Pleistocene *H. sapiens* individual Skhul V shows the most distinct overall shape relative to Hahnöfersand (position 44). At the same time, the most similar Middle Pleistocene European and Neanderthal are Sima de los Huesos 5 (position 19) and Shanidar 1 (position 21), respectively. The pattern observed based on the pairwise PDs is reflected in the Mahalanobis distances (MD) calculated between Hahnöfersand and the four group means of our comparative samples (Supplementary Table S3). Hahnöfersand is closest to the mean of the Holocene *H. sapiens* sample. Both the MDs to the Neanderthal and the Late Pleistocene *H. sapiens* group means are one order of magnitude larger, while the distance to our Middle Pleistocene European group mean is three orders of magnitude larger.

## Discussion

Our morphological re-evaluation of the frontal bone from Hahnöfersand is consistent with its revised Mesolithic date^22^. Multivariate analyses show a clear and unequivocal morphological affinity between Hahnöfersand and *H. sapiens* (Fig. 3, Table 3, Supplementary Table S3). The reported mix of *H. sapiens* and Neanderthal-like features in Hahnöfersand^16^ did not in any way affect its clear association with *H. sapiens* and did not result in an intermediate position of this specimen in our PCA (Fig. 3), as would be expected for an individual with admixed ancestry (e.g.,^44,51^).

The same overall result can be hypothesized for a completely preserved frontal bone from Hahnöfersand. The only commonly described variable not captured by our dataset is supraorbital thickness. Despite the common description, objective measurements are rare. Supraorbital thickness separates *H. sapiens* from Neanderthals and Middle Pleistocene Europeans, which exhibit, on average, thicker supraorbital regions, while not separating the latter two groups (e.g.,^38,40,52,53^). Therefore, including supraorbital thickness as a variable in our PCA would likely reinforce the separation between the *H. sapiens* and Neanderthal samples, with little effect on the relative positions of the Middle Pleistocene Europeans to the Neanderthal sample. For *H. sapiens* and Neanderthals, previous studies have shown that the medial region is, on average, the thickest (e.g.,^38,40^). The difference in thickness between the medial and lateral supraorbital region is more pronounced in *H. sapiens*. Although the actual supraorbital thickness in Hahnöfersand cannot be measured, due to its preservation, the clearly delimited superciliary region and partially preserved supraorbital trigone of Hahnöfersand suggest that the lateral supraorbital region would be significantly thinner than the medial one (cf. Fig. 2a, c, e), which would align with the pattern observed in *H. sapiens*.

The hypothesis that Hahnöfersand exhibits a more extreme morphology than average for the Mesolithic^22,54^ could not be tested directly due to the size of our Mesolithic comparative sample (N=3). However, all three Medieval *H. sapiens* closest in overall shape to Hahnöfersand (cf. Table 3) show a relatively average frontal bone morphology. Similarly, those individuals from our comparative sample exhibiting visually relatively robust frontal bone morphologies (Hohlenstein 5830 A, Drigge, Groß Fredenwalde 3, Heidelberg), which in the past may have been mistaken as extreme morphologies or Neanderthal-like, all plot within the Holocene *H. sapiens* variation and away from the Neanderthal sample (Fig. 3). Therefore, our results suggest that the frontal bone morphology of Hahnöfersand may not be as extreme as previous visual observations and qualitative data implied (cf.^16,22,54^).

The apparent contradiction between visual observations and qualitative data with the quantitative data presented in this study can be explained by a combination of three factors: 1) the influence of surrounding structures, 2) orientation of isolated fragments, and 3) intraspecific variability. The glabellar region is an example of the influence of surrounding structures on the qualitative interpretation of the supraorbital region. Athreya^55^ demonstrated that based on linear measurements, Neanderthals and *H. sapiens* show similar levels of projection in the glabellar region. However, the glabellar region of Neanderthals can be perceived as depressed, and that of *H. sapiens* as projecting or inflated (e.g.,^56–59^). Assessment of glabellar morphology, particularly visually, is influenced by the prominence of the superciliary regions as well as the form of the ascending portion of the frontal squama (e.g.,^38,55,60^). As a result, typical *H. sapiens* features in the supraorbital region can appear Neanderthal-like in overall robust individuals.

Furthermore, the interpretation of the ascending portion and, thereby, the midsagittal outline of the frontal squama are highly dependent on the orientation of the frontal bone. An orientation along a reference plane, e.g., the eye-ear plane (Frankfurter horizontal), might not be possible in fragmentary remains. Bräuer^16^ himself pointed out uncertainties in the correct orientation of Hahnöfersand in the alignment with comparative samples. These uncertainties might explain the absence of a proposed Neanderthal-like midsagittal outline in Hahnöfersand in our analyses (cf. Fig. 3 PC1;^16^), which relies on a Generalized Procrustes Analysis (GPA) for the proper comparison of all individuals (e.g.,^61^).

Moreover, our samples show considerable intraspecific variation. In the case of *H. sapiens*, this variation could be detected for individual traits (cf. Fig. 3) and overall shape. Skhul V is the *H. sapiens* individual most different from Hahnöfersand based on PD (Table 3) and known for its relatively low, receding frontal bone with a broad, more robust, projecting supraorbital region (e.g.,^37,55,62^). This intraspecific variation can only partially be explained through static allometry (Fig. 4, Table 2), while the geological age appears to play a more critical role. There is a tendency for geologically older *H. sapiens* (Skhul V, Qafzeh 9) to express more prominent supraorbital regions than Holocene *H. sapiens* (Fig. 3a). The Late Pleistocene sample associated with the Upper Paleolithic shows an intermediate morphology, with one-half of the individuals overlapping with the Holocene variation and the other half showing relatively prominent supraorbital regions similar to the geologically older Qafzeh 9. This is consistent with previous findings (e.g.,^43,44,51^), where Upper Paleolithic specimens consistently plot intermediate between Neanderthals and recent modern humans in various aspects of their cranial anatomy (e.g., Mladeč 5; Předmostí 3). Such a result may in part be associated with Neanderthal ancestry: all Upper Paleolithic Europeans for whom ancient DNA has been obtained show varying degrees of Neanderthal ancestry, which may influence cranial shape even after many generations, especially when Neanderthal alleles relevant for the expression of skeletal features are present^51,63^. Furthermore, although no genetic material has been recovered from the Skhul or Qafzeh remains, a possible admixed status has been previously hypothesized for these populations (e.g.,^64–66^), although their robust morphology may also represent retention of ancestral morphology in the *H. sapiens* lineage (e.g.,^51^; see also^67^).

Due to sample availability, most Upper Paleolithic individuals in our sample are associated with the Gravettian (cf. Supplementary Table S1). Recent paleogenetic evidence suggests two genetic clusters in European individuals associated with the Gravettian: the Věstonice cluster, including individuals from central–eastern and southern European sites, and the Fournol cluster, comprising individuals from western and southwestern European sites^68^. However, the observed distribution in frontal bone morphology does not match these suggested genetic clusters, as central-eastern European individuals plot both close to the geologically older individuals and in the Holocene variation. In contrast, the distribution might be explained by sexual dimorphism with (potential) females (Mladeč 1:^69^; Předmostí 4:^70^) showing a less prominent supraorbital region than (potential) males (Cioclovina:^71^; Mladeč 5:^69^; Předmostí 3:^70^). Sexual dimorphism was expected for the Pleistocene (e.g.,^40^) and Holocene samples (e.g.,^48,72^). However, inconclusive sex estimations for large parts of our sample hindered a clear evaluation. This includes key fossils in our sample, like Qafzeh 9 (cf.^73,74^) and Hahnöfersand (cf.^16,30^). Previous studies focusing on the supraorbital and upper facial regions are inconsistent and difficult to compare concerning potential temporal trends within *H. sapiens* (e.g.,^55,62,75^). A targeted study would be necessary to evaluate the influence of sexual dimorphism and geographic variation on the observed temporal intra-specific variation in *H. sapiens*.

In contrast, our Neanderthal sample reflects a relatively distinctive morphology (cf. Fig. 3), consistent with previous findings (e.g.,^12,43–46,51,62,76^). The Neanderthals plotting closest to the *H. sapiens* variation originate from Shanidar (e.g.,^55,62^) and show great similarities to individuals from Qafzeh, here Qafzeh 9 (e.g.,^55^), while the other West Asian Neanderthal from Amud clusters with the European Neanderthals (e.g.,^55,77,78^). The partially reconstructed Shanidar 1 and 5 have been described as showing a relatively vertical frontal squama, therefore a more *sapiens-*like morphology (e.g.,^62,78,79^), indicating some intraspecific variation in these features. This is consistent with their position along PC1, where both Shanidar individuals show more negative scores than other Neanderthal specimens, overlapping with Skhul V and plotting close to some of the other Pleistocene *H. sapiens* individuals (Fig. 3), but are clearly distinct from Holocene *H. sapiens*.

Studying overall morphology can provide an alternative for the study of isolated, fragmentary fossil remains, where contradicting results between individual traits can have a more considerable influence on assumed taxonomic affinities than in well-preserved, more complete remains. The toolkit of landmark-based geometric morphometrics allows the comprehensive study of a fossil’s shape by preserving its geometry throughout the analysis (e.g.,^61,80,81^). In the case of Hahnöfersand, the calculated intra- and inter-observer errors in landmark placement exceeded acceptable limits and precluded the use of traditional landmark-based geometric morphometrics (Supplementary Figure S6a; e.g.,^82^). The error was exceptionally high for ophryon, a landmark whose placement is influenced by the orientation of the frontal bone (for landmark definition, see Supplementary Table S2). However, landmarks routinely used, like bregma, also showed inconsistencies in placement between observers.

Another option for the study of fragmentary remains is the surface registration method. The previous application of this method to the hominin fossil record showed a significant reduction in intra- and inter-observer error compared to the error in landmark placement^49,50^. In a dataset with errors in landmark placement of ca. 0.1 to 1.1 mm, the average error between corresponding mesh vertices was reduced to ca. 0.03 mm. Similarly, meshes of Hahnöfersand created via the surface registration method based on the repeatedly placed landmarks exhibited an average error of ca. 0.05 mm (cf. Supplementary Figure S6d, Materials and Methods: Error Calculations).

Introducing a subsampling step to the surface registration method to reduce the amount of data used in subsequent statistical analyses adds an additional source of error (cf. Material and Methods). K-means clustering as the chosen subsampling method is naïve to the measurement error resulting from the initial landmark placement (e.g.,^83^). Further, k-means clustering leads to a discrepancy and loss of information when substituting the k centroids for the actual observations (e.g.,^84^). In our dataset, a subsampling from 28,700 vertices to less than 100 led to a visible discrepancy in the PCA plots (Supplementary Figure S3b). In addition, some datasets with more vertices, i.e., 200-500 vertices, also show a higher discrepancy. This can be explained by the position of the centroids during k-means clustering. The centroids are equally distributed on top of the original surface (e.g.,^85–87^), which, depending on their number, might not include surface structures driving the overall patterns of shape variation captured in a dataset without subsampling. Here, subsampling to 100 vertices slightly increases the average error between corresponding mesh vertices to 0.12 mm while remaining representative of the overall patterns of shape variation (Figure 3a, Supplementary Figures S3, S6a, e). With and without subsampling, the error is concentrated around the edges of the mesh (Supplementary Figures S6b, c), which can be explained by the high error in landmark placement of up to 3.08 mm (Supplementary Figure S6a). Therefore, the outermost edge of the region of interest should be considered with caution when interpreting the results, e.g., shape changes along PCs.

All in all, the error tests for Hahnöfersand confirmed the ability of the surface registration method to be robust against a substantial data reduction due to subsampling and to buffer uncertainties in landmark placement due to the exclusion of these landmarks in the final dataset. Nevertheless, a careful case-by-case evaluation of the intra- and interobserver error is required for future applications in cases with relatively high errors in landmark placement.

## Conclusion

Our results show that the frontal bone from Hahnöfersand, Germany, is most similar to Holocene *H. sapiens,* consistent with its revised Mesolithic date. Hahnöfersand does not exhibit an intermediate morphology between Neanderthals and *H. sapiens,* contrary to previous assessments of its morphology.

Furthermore, the perceived extreme frontal bone morphology of Hahnöfersand and other Holocene *H. sapiens* might be at least in part the effect of visual, qualitative observations and the difficulty in properly aligning incomplete specimens. In combination with the potential temporal trend and sexual dimorphism observed in our *H. sapiens* sample, this intragroup variation warrants further exploration. Understanding variation, both in Holocene *H. sapiens* and in fossil groups, is the basis for secure taxonomic identification of fragmentary remains that lack a secure context and/or dating. Especially isolated fragments associated with the Late Pleistocene in Europe or other regions and time periods where (potentially) multiple hominin groups were present penecontemporaneously require careful examination and, in the absence of ancient DNA, should be reevaluated regularly, employing state-of-the-art knowledge of variation and methodologies.

Here, we demonstrated the usefulness of the surface registration method for the morphological assessment of taxonomy in fragmentary remains and introduced an open-access function, including a subsampling option, for applying this novel approach in the open-source R software environment.

## Materials and methods

### Sample composition

We evaluated the external morphology of Hahnöfersand through a set of 44 surface models extracted from Computed Tomography (CT) and surface scans of Middle Pleistocene (~MIS 12-7) pre-Neanderthals from Europe, *H. neanderthalensis* (MIS 7-3), and a diverse *H. sapiens* sample (Table 1, Supplementary Table S1). The latter was further subdivided into Late Pleistocene *H. sapiens* (~MIS 5-2) and Holocene *H. sapiens*. These samples include many of the individuals used in the original comparative analysis of Hahnöfersand (cf.^16^). In addition, the Holocene *H. sapiens* sample contains individuals that either show relatively prominent supraorbital regions, e.g., Heidelberg, Hohlenstein 5830 A, and Groß Fredenwalde 3, or supposed morphological similarities to Hahnöfersand, e.g., Drigge (cf.^16,22^).

### Hahnöfersand surface model and reconstruction

The Hahnöfersand frontal bone (HMV 80.18) is housed at the Archaeological Museum in Hamburg, Germany. In the context of the re-dating mentioned above, a surface scan was acquired before removing the dating sample. Fig. 2c and d show the state of preservation in the early 2000s before the sampling, while today, the missing aspect of the right frontal squama is more extensive (Fig. 2a, b). It was possible to utilize the more complete digital record of Hahnöfersand due to the relatively good quality of the existing surface model (90,437 vertices; 180,870 faces with an average edge length of 0.64 mm).

This surface model made it possible to reconstruct Hahnöfersand further (cf. Fig. 2e, f, Supplementary Figure S2). Therefore, a midsagittal plane was defined through the glabella, bregma, and foramen caecum, along which both sides of the surface model were mirrored. The positions of both mirrored sides were adjusted to match the preserved, slightly asymmetrical morphology in the Hahnöfersand frontal bone. The resulting reconstruction includes a complete frontal squama, bilateral temporal lines between the respective frontotemporale and stephanion, and a relatively complete supraorbital region (Supplementary Figure S2).

### Measurement protocol

Attempts to analyze the ancient DNA (aDNA) of Hahnöfersand were unsuccessful. The amount of preserved endogenous DNA was too small for conclusive results (pers. com. C. Posth). Thus, we focused our analysis on the surface registration method. Surface registration is an almost landmark-free approach that allows us to study the entire preserved morphology (for discussion, see^49,50^). A reference mesh, e.g., of the preserved external morphology of the fossil in question, is deformed to best match a set of target meshes using Gaussian-smoothed displacement vectors (e.g.,^88,89^). A sample of triangular meshes with an identical number of corresponding vertices is created, which can be extracted and used as coordinates during analyses.

In the first step, single-layered triangular meshes were extracted from the CT and surface scans. For the CT scans, surface models were generated via semi-automated segmentation using threshold-based segmentation in all slices, followed by local manual corrections. In cases where these surface models and models created via surface scanning were virtually reconstructed, like Hahnöfersand, the surface models require a remeshing before creating single-layered meshes (e.g., in Blender version 4.4.3 via object mode::modifier::remesh) to remove all internal overlapping mesh structures and to create a mesh that cannot be split into multiple connected components. Subsequently, all models were transformed into single-layered triangular meshes of the region of interest, here the external table of the frontal bone, by removing the residual mesh vertices. Next, six landmarks were placed on each mesh (Fig. 2e, f; Supplementary Table S2). Thereby, the length of the mesh is approximated by the chord between the midsagittal landmarks bregma (1) and ophryon (2), the breadth by the chords between the bilateral frontotemporale (3 & 4) and stephanion (5 & 6), respectively, and the length of the bilateral temporal lines as chord between frontotemporale (3/4) and stephanion (5/6).

In the third step, a TPS algorithm was used to perform a landmark-based registration between the reference, here Hahnöfersand, and every mesh in the comparative sample (e.g.,^61^). This was followed by an iterative closest point (ICP) matching in which an elastic ICP algorithm calculates the best match of Hahnöfersand onto each mesh in the comparative sample (for a detailed discussion, see^49,50^).

Contrary to the initial publication of the surface registration method (cf.^49,50^), the entire third step was carried out as a single R function for the whole dataset. This simplifies and significantly accelerates the use of the surface registration method. In addition, the new R function allows subsampling to reduce the data and variables for subsequent analyses. The subsampling function is based on a k-means clustering algorithm. The k-means method aims to partition the data, here the coordinates underlying the vertices in each mesh, into k groups such that the sum of squares from each vertex to the assigned cluster center is minimized (e.g.,^85–87^). At the minimum, all cluster centers are at the mean of their vertex sets. Next, Euclidean distances between the cluster means and their surrounding vertices were calculated. For each cluster, the vertex with the smallest Euclidean distance to the cluster mean was included in the subsample of vertices. This step ensures that the subsample consists of actual surface vertices rather than artificially calculated points. The output of the R function is either a 3-dimensional array containing the coordinates underlying the non-subsampled vertices or a subsample thereof for all meshes in the dataset.

As a fourth and final step, GPA was used to superimpose the extracted coordinates to allow for statistical analysis of the resulting Procrustes shape coordinates. During GPA, information about location and orientation is removed, and the landmark configurations are scaled to a standardized size (i.e., centroid size, e.g.,^61^).

### Determining the optimal subsample

We ran multiple iterations of the above-described surface registration on our dataset to determine the ideal subsample. The first iteration without subsampling was considered as a reference. An additional 32 iterations were calculated, 16 without subsampling to create a baseline and 16 with subsampling, ranging from 25 to 5k vertices. For each iteration, we performed a Principal component analysis (PCA). Based on the Principal Component (PC) scores, distance matrices were calculated and converted into vectors. Each vector originating from one of the additional iterations was compared to the reference iteration via a Pearson correlation test (Supplementary Figure S3). A Pearson correlation coefficient (r) visually deviating from the baseline created by the iterations without subsampling is considered to affect the PCA results. Therefore, the smallest subset showing a correlation coefficient similar to the baseline is here regarded as an optimal subsample for maximal data reduction without substantial information loss during the k-means clustering (cf. Supplementary Figure S3). Thereby, the optimal subsample is highly dependent on the discriminating power of individual features and their size. For example, for a surface with relatively small features that discriminate within a given sample, the optimal subsample will be relatively large. As a consequence, the optimal subsample should be evaluated on a case-by-case basis to ensure the dataset is appropriate for the given research question.

### Analyses

Patterns of shape variation were investigated via PCA of the shape variables for a dataset with subsampling to 100 vertices. An additional PCA was calculated in which Hahnöfersand was not used to calculate the PCA but was instead projected into the plots, following established procedure (see, e.g.,^11,12,90,91^). Shape changes occurring along each plotted PC were illustrated as a visualization of the vertices of each PC at ± 2 standard deviations. Groups and species attribution were not part of the calculation of the PCA, which is independent of group membership, but were shown post facto in the form of convex hulls in the corresponding PCA plots. Convex hulls were calculated around the extreme points of each defined group and contained no information about confidence intervals.

In addition, allometry was assessed on the level of static allometry, i.e., the correlation of shape and size independent of biological age. Size was measured as surface area and shape in the form of the first three PCs from the dataset, with subsampling to 100 vertices and without projection of Hahnöfersand. Each PC was plotted against size to visualize its allometric relationship; the regression slope and residual standard error were calculated, and its correlation was tested via Pearson correlation tests. Statistical tests were considered significant at α ≤ 0.05.

Overall shapes between individuals were compared based on Procrustes Distances (PD) of the entire shape captured in the Procrustes superimposed vertex coordinates of the dataset (e.g., ^43,92^). The PD mean and standard deviation were calculated for each group as summary statistics. In addition, Mahalanobis distances (MD) between Hahnöfersand and the group means of the four comparative samples were computed. For each group, the mean shape and covariance matrix were calculated from the Procrustes-superimposed vertex coordinates of all individuals assigned to that group.

### Error calculations

Multiple measurements of the same individual, i.e., Hahnöfersand, were carried out by three observers (C.R., M.B., M.S.). Two observers (M.B., M.S.) were unaware of the research question and were only provided with a 3-dimensional surface model and the landmark definitions (Supplementary Table S2). Each observer collected repeated measurements over a period of several weeks.

Distances between repeated measures of the landmarks placed for initial registration and between resulting surfaces were calculated. Landmarks for initial registration were evaluated via Euclidean distances in mm between repeated measures of the individual landmark positions (e.g.,^82^). The intra- and inter-observer errors range from 0.19 to 3.08 mm (Supplementary Figure S6a). Further, mesh distances were used to evaluate the effect of the error in landmark placement on the output of the surface registration method. Therefore, the surface registration method was run with and without subsampling using the repeatedly measured landmarks of all three observers and their corresponding mesh as the target dataset. Distance heat maps between the computed meshes illustrate that the displacement exceeding 1 mm is confined to the edges of the meshes and can be explained by the observer error during landmark placement (cf. Supplementary Figures S6b, c). In contrast, almost no displacement can be detected within the surface area. Out of all mesh vertex combinations of the error measurements, only 0.87% (673 out of 77,355) and 2.4% (36 out of 1,500) exceeded a displacement of 1 mm without and with subsampling, respectively (Supplementary Figures S6d, e).

The results of the error calculations are discussed in detail in the second half of the Discussion section.

### Software

All steps of the measurement protocols and the following analyses were carried out in a combination of four different software environments. Avizo 3D 2023 (Thermo Fischer Scientific) was used to extract meshes from CT scans and to obtain landmarks. Blender (version 4.4.3;^93^) was used to remesh all reconstructed meshes, including the refence individual. Meshlab (Visual Computing Lab of ISTI-CNR) was used to transform meshes into single-layered triangular meshes. All other steps were carried out in R^94^ by using freely available R packages, mainly geomorph (version 4.0.7;^95^), mesheR (version 0.4.200213;^96^), Morpho (version 2.12;^97^), Rvcg (version 0.22.2;^97^), Arothron (version 2.0.5;^98^), and shapes (version 1.2.7;^99^), plotly (version 4.12.0;^100^). The R function introduced here for the surface registration combines functions from the packages mentioned above, especially Morpho, and is freely available via https://github.com/CaroRoeding/surfreg. Graphics were created in R and processed in Adobe Illustrator CS5.

## Supplementary Information

Supplementary Information.

## Data Availability

The triangular mesh surface of Hahnöfersand is available for non-commercial use at Zenodo. The R function is available for non-commercial use at https://github.com/CaroRoeding/surfreg. In both cases, the current study acts as a reference and should be cited for every use. The comparative data presented in the current study are available from their respective housing institutions/repositories upon reasonable request.
