# Supplementary material for: A morphological analysis of the modern human frontal bone from Hahnöfersand, Germany
Source: Sci Rep. 2026 Apr 17;16:12696. doi: 10.1038/s41598-026-48468-5 (PMC13090399; doi:10.1038/s41598-026-48468-5)
Supplement: Supplementary file 1 — Supplementary Information. [file 41598_2026_48468_MOESM1_ESM.pdf]

A morphological analysis of the modern human frontal bone from Hahnöfersand, Germany

Carolin Röding, Antonio Profico, Michael Merkel, and Katerina Harvati

Table of Contents

Supplementary Tables **2**

Supplementary Table S1: Detailed sample composition. 3

Supplementary Table S2: Landmark definitions 4

Supplementary Table S3: Mahalanobis distances (MD) between Hahnöfersand and the group means of our comparative samples 5

Supplementary Figures **6**

Supplementary Figure S1: Surface model of the frontal bone from Hahnöfersand 7

Supplementary Figure S2: Surface model of the virtually reconstructed frontal bone from Hahnöfersand 8

Supplementary Figure S3: Visualization of the effect of the subsampling step during the surface registration method 9

Supplementary Figure S4: Shape PCA with subsampling to 100 vertices and Hahnöfersand projected 10

Supplementary Figure S5: Interactive 3d scatter plot of the first three PCs 11

Supplementary Figure S6: Visualizations of intra- and inter-observer error calculations.. 12

References Supplementary Material **13**

Supplementary Tables

Supplementary Table S1: Detailed sample composition. Abbreviations for figures only provided for individuals dating to the Pleistocene or for which previously an unusual frontal bone morphology was described.

Species	Groups based on dating / associated archaeology		Country	Individuals	Abbreviations	Access provided by
Homo sapiens	Holocene	Mesolithic	Germany	Hahnöfersand HMV 80.18	Ha	Archäologisches Museum Hamburg, Germany
				Drigge	Dr	Stralsund Museum, Germany (C. Hoffmann)
				Groß Fredenwalde 3	GF3	Landesamt für Denkmalpflege Brandenburg, Germany (A. Kotula)
				Hohlenstein 5830 A (male)	Ho	osteological collection Tübingen, Germany (H. Rathmann)
		Neolithic (Linear Pottery Culture)		Schwetzingen 4, 5, 14, 43, 48, 67, 101, 109, 119, 122<br>Talheim 83_8, 83_12_2, 83_22_E, 84_2		Landesamt für Denkmalpflege Baden-Württemberg, Germany (M. Francken, A. Spatzier, D. Krausse)
		Medieval (ca. 600-900 AD)		Nusplingen 1106a, 1106b, 1111a, 1119, 1122, 1124, 1125		osteological collection Tübingen, Germany (H. Rathmann)
				Heidelberg HD-Ber 2010/12	He	Kurpfälzisches Museum Heidelberg, Germany (T. Schöneweis, late R. Ludwig)
	Late Pleistocene		Romania	Cioclovina	Ci	Department of Geology, University of Bucharest, Romania
			Italy	Grotte des Enfants 6	GdE6	E. Delson (NYCEP)
			Czech Republic	Mladeč 1	Ml1	EVAN Society, Vienna, Austria
				Mladeč 5	Ml5	E. Delson (NYCEP)
				Předmostí 3	Pr3	
				Předmostí 4	Pr4	
			Israel	Qafzeh 9	Qa9	
				Skhul 5	Sk5	Peabody Museum, Harvard, USA
Homo neanderthalensis	Late Pleistocene			Amud 1	Am1	E. Delson (NYCEP)
			Italy	Guattari 1	Gu1	
			France	La Chapelle	LC	
				La Ferrassie	LF	
			Germany	Neandertal	Ne	
			Iraq	Shanidar 1	Sh1	
				Shanidar 5	Sh5	
			Belgium	Spy 1	Sp1	NM digital archive
Middle Pleistocene Europe			France	Arago 21	Ar21	E. Delson (NYCEP)
			Spain	Sima 5	Si5	
			Greece	Petralona	Pe	Paleontological Museum, University of Thessaloniki, Greece (D. Kostopoulos)

Supplementary Table S2: Landmark definitions for the initial registration of the surface registration method. Visualization provided in Supplementary Figure S6.

Number in Figure	Position of the landmark	Landmark Definition[1]
1	midsagittal	bregma (b): the point at the intersection of the sagittal and coronal sutures
2		ophryon (on): point at the intersection of the midsagittal plane and the best fit plane of the frontoemporalia and orbital roofs; usually falls into the upper glabellar region
3 & 4	bilateral	frontotemporale (ft): the most anterior and medial point of the superior temporal line
5 &6		stephanion (st): the point at the intersection of the coronal suture and superior temporal line

[1]following the definitions by [1]

Supplementary Table S3: Mahalanobis distances (MD) between Hahnöfersand and the group means of our comparative samples (Holocene *H. sapiens* N=25, Late Pleistocene *H. sapiens* N=8, *H. neanderthalensis* N=8, Middle Pleistocene Europeans N=3). MDs based on Procrustes coordinates of the subsampled dataset with 100 vertices. The smallest MD to Hahnöfersand is marked in bold.

Group mean	Mahalanobis Distance/(1e+16)[1]
Holocene *H. sapiens*	**-7.879**
Late Pleistocene *H. sapiens*	88.563
H. neanderthalensis	-50.544
Middle Pleistocene Europeans	4908.291

[1]rounded to three decimals

Supplementary Figures

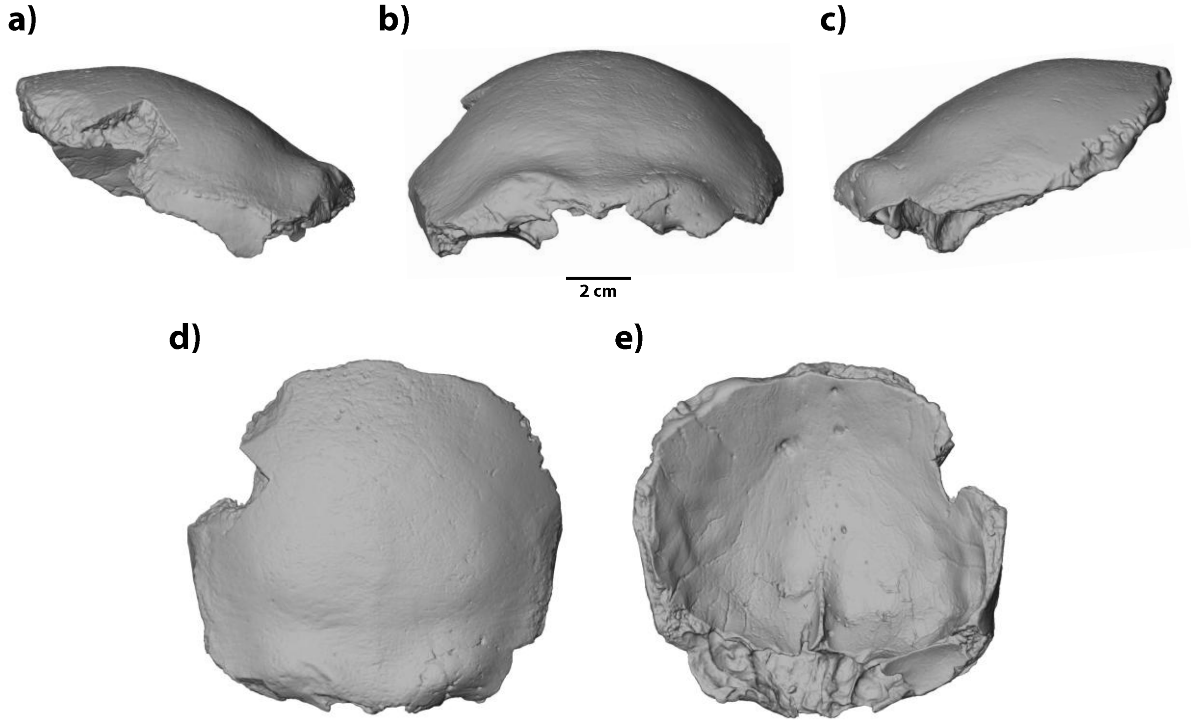

Supplementary Figure S1: Surface model of the frontal bone from Hahnöfersand rendered from the surface scan acquired in the early 2000s. The frontal bone is shown in lateral right view (a), anterior view (b), lateral left view (c), superior view (d), and inferior view (e).

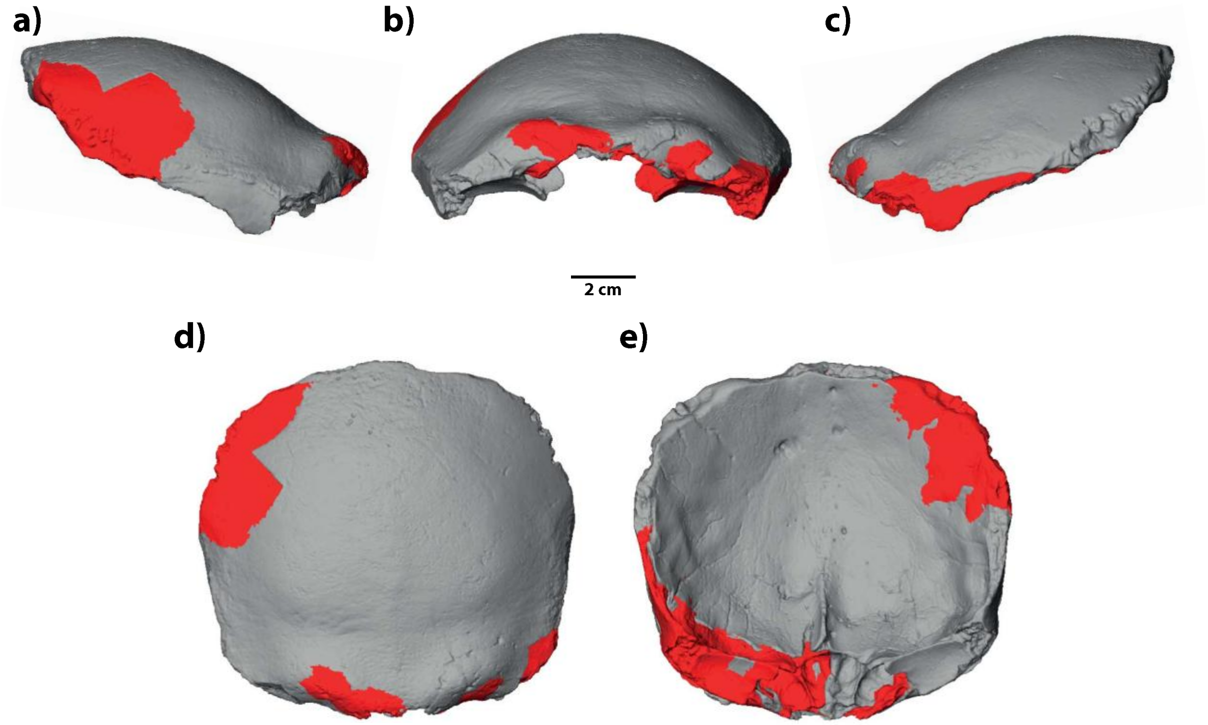

Supplementary Figure S2: Surface model of the virtually reconstructed frontal bone from Hahnöfersand with reconstructed elements shown in red. The frontal bone is shown in lateral right view (a), anterior view (b), lateral left view (c), superior view (d), and inferior view (e).

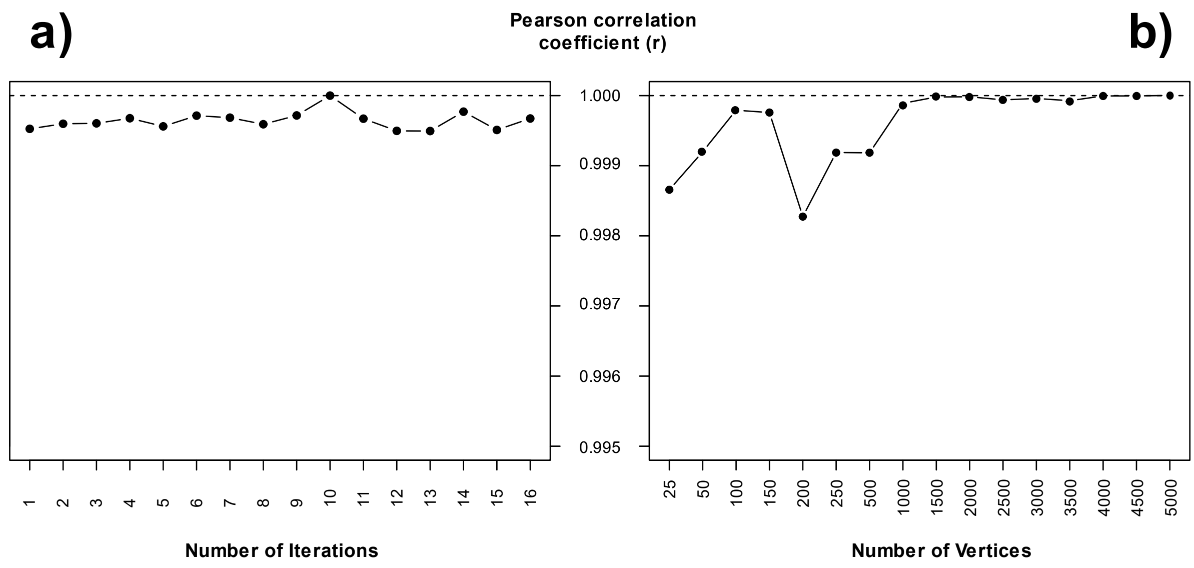

Supplementary Figure S3: Visualization of the effect of the subsampling step during the surface registration method on subsequent PCA results. A) Pearson correlation coefficients between distance matrices calculated from PCAs of 16 iterations without subsampling to that of the initial reference iteration. B) Pearson correlation coefficients between distance matrices calculated from PCAs of 16 iterations with different levels of subsampling to that of the initial reference iteration

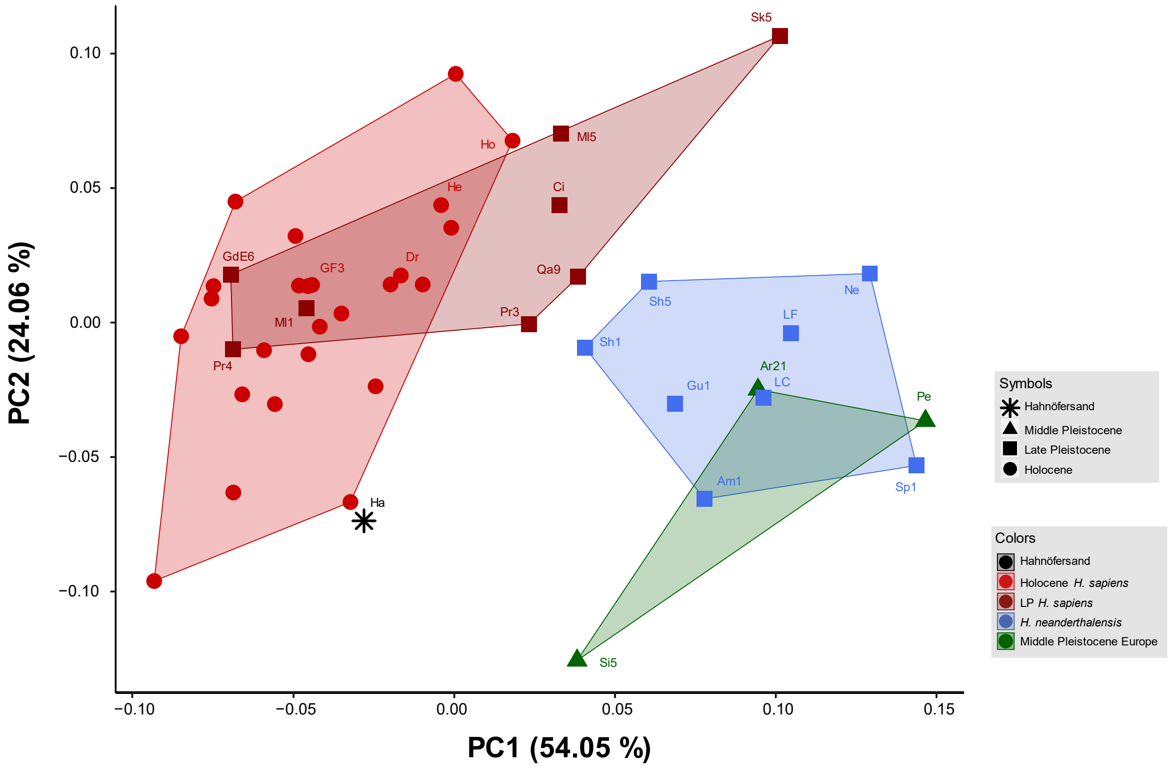

Supplementary Figure S4: Shape PCA with subsampling to 100 vertices and Hahnöfersand projected into the projection of PC1 against PC2. Shape changes along PC axes are illustrated as warped surfaces at the extremes of each PC. Information about the sample and abbreviations of all fossil individuals are listed in Tables 1 and Supplementary Table S1.

For the interactive model
(SOM2_scatterplot3d_PCA_Hahnöfersand.html)
please click into the box.

Supplementary Figure S5: Interactive 3d scatter plot of the first three PCs of a shape PCA with subsampling to 100 vertices. Due to the nature of the interactive plot, there are no shape changes along PC axes displayed; for their illustrations, see Figure 3. Information about the sample and abbreviations of all fossil individuals are listed in Tables 1 and Supplementary Table S1. This plot was created with the R package plotly [2].

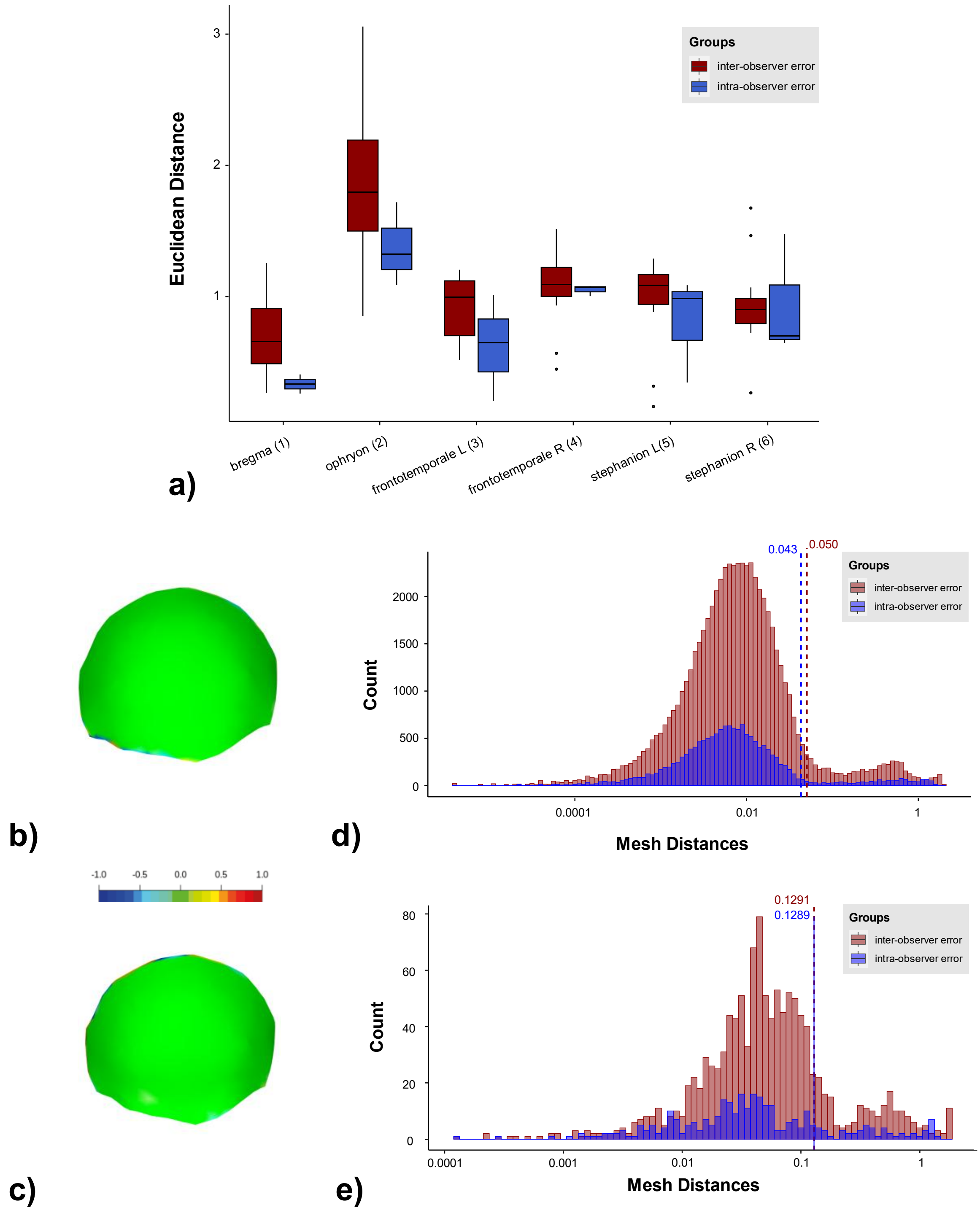

Supplementary Figure S6: Visualizations of intra- and inter-observer error calculations. A) Box-Whisker plots of pairwise Euclidean Distances in mm illustrate the error in placing the landmarks for initial registration. B) Example distance heat maps between meshes created via surface registration without subsampling and C) with subsampling to 100 vertices. Distances in mm are translated to a color code ranging from no difference in green to ≥ 1 mm difference in dark red and≥ -1 mm difference in dark blue. D) Histogram of distances in mm between meshes without subsampling and E) with subsampling to 100 vertices. Distances are shown as log-transformed on the x-axis, and dotted lines indicate group means.

References Supplementary Material

1 Martin, R. & Saller, K. *Lehrbuch der Anthropologie in systematischer Darstellung mit besonderer Berücksichtigung der anthropologischen Methoden*. 3rd edn, Vol. 1 (Fischer, 1957).

2 Sievert, C. Interactive Web-Based Data Visualization with R, plotly, and shiny. (2020). < https://plotly-r.com>.
